# Supplementary material for: Molecular and Hormonal Aspects of Drought-Triggered Flower Shedding in Yellow Lupine
Source: Int J Mol Sci. 2019 Jul 31;20(15):3731. doi: 10.3390/ijms20153731 (PMC6695997; doi:10.3390/ijms20153731)
Supplement: Supplementary file 1 [file ijms-20-03731-s001.pdf]

**Acronyms used in the text**

| **ABBREVIATIONS** in alphabetical order | **DEFINITION** |
|---|---|
| **ABA** | abscisic acid |
| **ACC** | aminocyclopropane-1-carboxylic acid |
| **AZ** | abscission zone |
| **CAT** | catalase |
| **DIST** | flower pedicel fragments above the az |
| **ET** | ethylene |
| ***LlACO*** | *AMINOCYCLOPROPANE-1-CARBOXYLIC ACID OXIDASE from Lupinus luteus* |
| ***LlACS*** | *AMINOCYCLOPROPANE-1-CARBOXYLIC ACID SYNTHASE from Lupinus luteus* |
| ***LlHSL*** | *RECEPTOR-LIKE PROTEIN KINASE HAESA-like from Lupinus luteus* |
| ***LlIDL*** | *INFLORESCENCE DEFICIENT IN ABSCISSION-like from Lupinus luteus* |
| ***LlMPK6*** | *MITOGEN-ACTIVATED PROTEIN KINASE6 from Lupinus luteus* |
| ***LlZEP*** | *ZEAXANTHIN EPOXIDASE from Lupinus luteus* |
| **PROX** | stem fragments below the AZ |
| **RWC** | relative water content |
| **VB** | vascular bundles |
| **WHC** | water holding capacity |

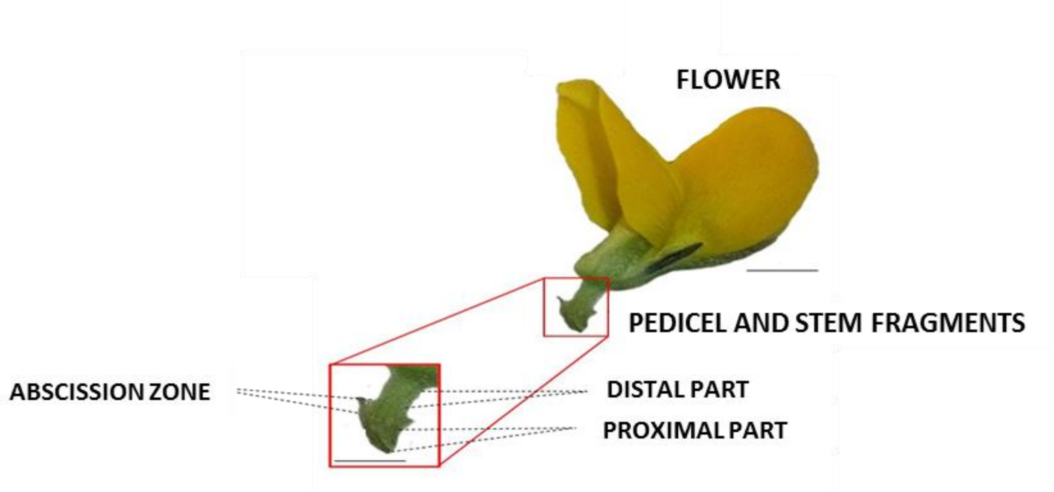


**Supplementary Figure 1.** Flower of *Lupinus luteus* L. Tissue fragments (indicated by red boxes) containing abscission zone (AZ) dissected 1 mm above (distal part) and below AZ (proximal part) were collected for analyses. Scale bars: 5 mm.

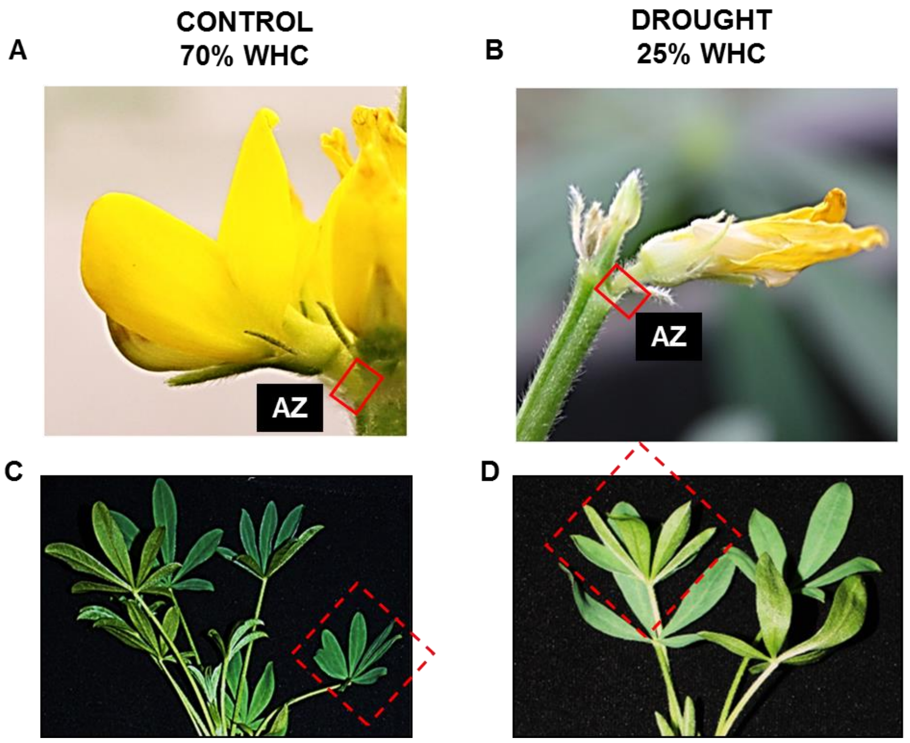


**Supplementary Figure 2.** Non-abscised flower of yellow lupine (*Lupinus luteus* L.) with inactive abscission zone (**A**) and flower containing active AZ from plants cultivated under drought conditions (**B**). The AZ regions were marked by red squares. Leaves of control plants (**C**) and leaves from drought-stressed lupines (**D**) used for analyses.

**Supplementary Table 1.** Qualitative and quantitative composition of calibration standards used in the ICP-OES analyses.

| | Concentration [ppm] | | |
|---|---|---|---|
| | **I** | **II** | **III** |
| **P** | 5 | 15 | 30 |
| **Fe** | 0.25 | 1.5 | 4 |
| **S** | 2 | 8 | 15 |
| | **IV** | **V** | **VI** |
| **Na** | 2 | 15 | 30 |
| **K** | 5 | 20 | 40 |
| **Zn** | 0.1 | 0.7 | 1.5 |

**Supplementary Table 2.** List of wavelengths characterized for measurements of emission lines of elements.

| Chemical element | Wavelength [nm] |
|---|---|
| Zn | 206.201 |
| | 213.857 |
| Na | 589.585 |
| | 588.998 |
| K | 766.496 |
| P | 213.619 |
| | 214.919 |
| S | 180.67 |
| | 182.562 |
| Fe | 238.203 |
| | 239.57 |
| | 259.938 |

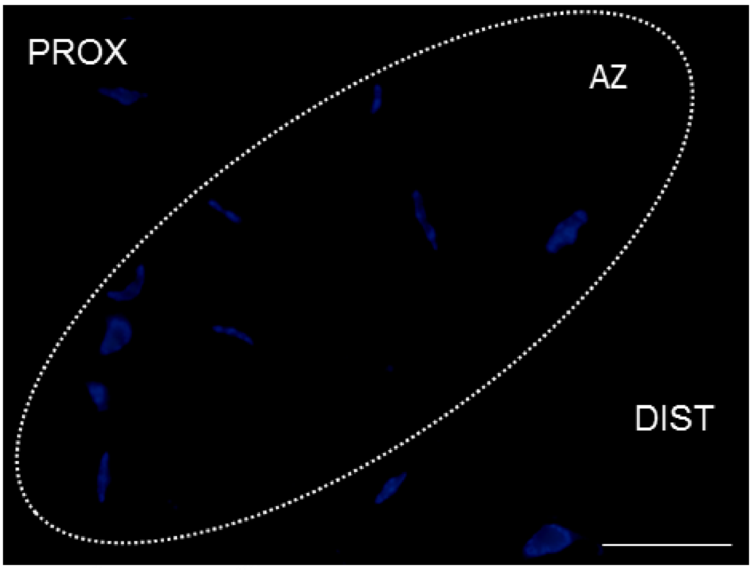


**Supplementary Figure 3.** The results of a control reaction performed with omitting primary antibodies anti-ACC, anti-ABA, anti-CAT, and anti-MPK6. Control reaction produced negative results compared with those of standard reactions. Nuclei were stained with DAPI. The abscission zone (AZ) region was highlighted by white curves. Abbreviations: PROX – stem fragment below the AZ, DIST – flower pedicel fragment above the AZ. Scale bars 100 μm.

**Supplementary Table 3.** List of primers used for identification of genes

| | cDNA of gene | Primer sequence 5'-3' |
|---|---|---|
| **Degenerate primers** | *LlZEP* | FP: GCTAYACTTGTTAYACYGGNA<br>RP: CCVACRCCHARRTAWGCYTT |
| **RACE-PCR primers** | *LlZEP* | FP: CATGCAGCCAAATATGGGCCAAGGA<br>RP: AATCACCGAGCAAAGTCACACGACC |
| **Specific primers** | *LlHSL* | 1FP: TCCCTCTCTCTAACCCAAGACG<br>1RP: CTCGTCCTGCAATGAGAAGGGTGG<br>2FP: TTGCAACTGGAAAGTGGCTCT<br>2RP: ATCACGAACAATTCCACCCGGA<br>3FP: GCAAGTTTGTGCAATGGGGGA<br>3RP:CCTAAGTATACGGGAAGCTCTG |
| | *LlMPK6* | 1FP: GCAACTTGTTCGTGCGTCTCGGA<br>1RP: GCAACTTGTTCGTGCGTCTCGGA<br>2FP: AGGGTTCTCACACATGGTGG<br>2RP: AGGGTTCTCACACATGGTGG |

**Supplementary Table 4.** List of primers and UPL probes used for RT-qPCR reactions

| cDNA of gene | Primer sequence 5'-3' | UPL no. |
|---|---|---|
| *LlIDL* | F: ACAGCACCATGGTCACTTTTC<br>R: TTGTGTTTCCTTGAGGGAGTG | 137 |
| *LlACS* | F: TTCATTCAAGAAGGCAATGGT<br>R: GGTTTGGGTCAAAAGTCACC | 53 |
| *LlACO* | F: GTGATGAAGGAATTTGCACAAG<br>R: CCAAGGTTTTCACACAGCAA | 53 |
| *LlZEP* | F: GGGTTTCCGGTTCTTGGTAT<br>R: AAAGCCATTCGACTAATAACTCTTG | 165 |
| *LlACT* | F: TAATGGTTGGGATGGGTCAG<br>R: CAAGGTGAGAATACCCCTCT | 165 |
| *LlHSL* | F: TGATGAAATTGGGTATTTGGGTA<br>R: GAAGCCGGTCAGAAAATTTATTATAG | 147 |
| *LlMPK6* | F: GCTCCTTCGTCATATGGATCA<br>R: AATGTAGACATCATTAAAGCTCTCCTT | 147 |

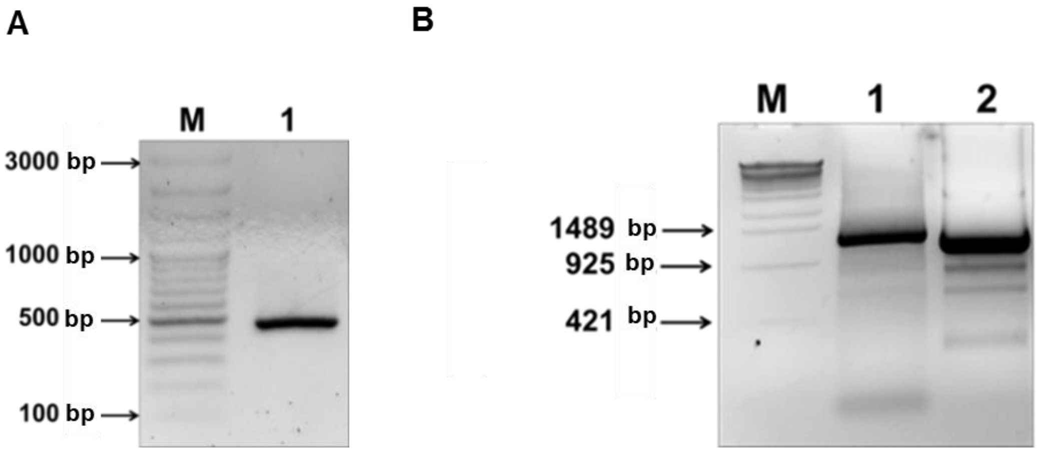


**Supplementary Figure 4.** Products of PCR with degenerate primers for *LlZEP* (**A**) and products of 5'-3'- RACE-PCR reactions (**B**; 1 and 2 lane, respectively) in agarose gel.

```
   1 atggggactttgttgccccttgtttttctatatcagatatattattagggacgaaaaaa
  62 ctcaacctctaacaatctttatcttcatttaaattattgcataccagttattggcttatg
 122 agtttggaaagttagagccactgaaagacacgtaacacaacacaacacacacttgagttt
 182 gttcctaagctttcttcttctgcaaccATGGCTTCTACCTTGTGTTACAATTCTCTTAAT
                                 M  A  S  T  L  C  Y  N  S  L  N   11
 242 ACCTCAACAACTTCTTTCTCAAGAACCCACTTCTCAGTTCCAATCAATAAAGAGCTTTCA
      T  S  T  T  S  F  S  R  T  H  F  S  V  P  I  N  K  E  L  S   31
 302 CTAGATATTTCACCTTTTGTTAGCTACGGCTATCACCTAGGAACCAGAACTAGGAAGCAG
      L  D  I  S  P  F  V  S  Y  G  Y  H  L  G  T  R  T  R  K  Q   51
 362 AAGAAGAAAGTGATGCTGCATGTAAAAGCTGGTGCAGTTGCTGAAGCTCCACCTTCAAAG
      K  K  K  V  M  L  H  V  K  A  G  A  V  A  E  A  P  P  S  K   71
 422 AAATCTGAGGCTGAAAATGGTGGGAATGGAATTACCCATCAGAAGAAGAAGCTTAGGATA
      K  S  E  A  E  N  G  G  N  G  I  T  H  Q  K  K  K  L  R  I   91
 489 CTTGTGGCAGGTGGTGGGATTGGTGGCTTGGTTTTTGCTTTGGCTGCTAAGAGAAAAGGG
      L  V  A  G  G  G  I  G  G  L  V  F  A  L  A  A  K  R  K  G  111
 542 TTTGAGGAGGTGGTGTTTGAGAGGGACTTGAGTGCTATAAGAGGTGAGGGACAGTATAGG
      F  E  E  V  V  F  E  R  D  L  S  A  I  R  G  E  G  Q  Y  R  131
 602 GGTCCAATTCAGATACAAAGTAATGCATTGGCTGCTTTGGAAGCTATAGATTCAGATGTT
      G  P  I  Q  I  Q  S  N  A  L  A  A  L  E  A  I  D  S  D  V  151
 662 GCTGATGAAGTTATGAGAATTGGTTGCATCACAGGTGATAGAATCAATGGACTTGTTGAT
      A  D  E  V  M  R  I  G  C  I  T  G  D  R  I  N  G  L  V  D  171
 722 GGGGTTTCCGGTTCTTGGTATGTCAAGTTTGATACATTCACTCCTGCAGTTGAACGTGGG
      G  V  S  G  S  W  Y  V  K  F  D  T  F  T  P  A  V  E  R  G  191
 782 CTTCCAGTCACAAGAGTTATTAGTCGAATGGCTTTACAAGAGATCCTTGCTAGTGCGGTT
      L  P  V  T  R  V  I  S  R  M  A  L  Q  E  I  L  A  S  A  V  211
 842 GGGGAAGATATCATCATGAATGGCAGTAATGTTGTAAATTTTGTAGATGATGGAAACAAG
      G  E  D  I  I  M  N  G  S  N  V  V  N  F  V  D  D  G  N  K  231
 902 GTAACAGTAGAGCTTGAGAATGGTGAGAAATATGAAGGAGATCTTTTGGTTGGAGCTGAT
      V  T  V  E  L  E  N  G  E  K  Y  E  G  D  L  L  V  G  A  D  251
 962 GGTATACGCTCCAAGGTGAGGAATCAGTTATTTGGTCCAAAAGAAGCTGTTTACTCCGGG
      G  I  R  S  K  V  R  N  Q  L  F  G  P  K  E  A  V  Y  S  G  271
1022 TATACTTGTTATACTGGTATTGCAGATTTTGTGCCTGCTGACATTGATTCTGTTGGGTAC
      Y  T  C  Y  T  G  I  A  D  F  V  P  A  D  I  D  S  V  G  Y  291
1082 CGAGTTTTCTTGGGACACAAACAATACTTTGTATCTTCAGATGTTGGTTCTGGAAAGATG
      R  V  F  L  G  H  K  Q  Y  F  V  S  S  D  V  G  S  G  K  M  311
1142 CAATGGTATGCATTTCACAAAGAAGCTCCTGGTGGTGTTGATATCCCCAATGGAAAAAAG
      Q  W  Y  A  F  H  K  E  A  P  G  G  V  D  I  P  N  G  K  K  331
1202 GAAAGGCTGCTTAGGATATTTGAGGGCTGGTGTGACAATGCAATAGATCTGATACTGGCT
      E  R  L  L  R  I  F  E  G  W  C  D  N  A  I  D  L  I  L  A  351
1262 ACAGACGAGACGGAAATTCTGCGACGAGACATCTATGACAGGATACCAACATTGAACTGG
      T  D  E  T  E  I  L  R  R  D  I  Y  D  R  I  P  T  L  N  W  371
1322 GGAAAGGGTCGTGTGACTTTGCTCGGTGATTCGGTCCATGCCATGCAGCCAAATATGGGC
      G  K  G  R  V  T  L  L  G  D  S  V  H  A  M  Q  P  N  M  G  391
1382 CAAGGAGGTTGCATGGCTATTGAGGACAGTTACCAACTAGCATTGGAGGTGGACAATGCA
      Q  G  G  C  M  A  I  E  D  S  Y  Q  L  A  L  E  V  D  N  A  411
1442 TGGGAGCAAAGTATAAAATCAGGCCAACTAGCATTGGAGGTGGACAATGCATGGGAGCAA
      W  E  Q  S  I  K  S  G  Q  L  A  L  E  V  D  N  A  W  E  Q  431
1502 AGTATAAAATCAGGGTCTCCGATTGACGTTGACTCTTCCCTTAGGAGCTACGAGAGAGAA
      S  I  K  S  G  S  P  I  D  V  D  S  S  L  R  S  Y  E  R  E  451
1562 AGAAGACTACGGGTTGCCATAATTCATGGAATGGCTAGAATGGCCGCTCTGATGGCTTCC
      R  R  L  R  V  A  I  I  H  G  M  A  R  M  A  A  L  M  A  S  471
1622 ACCTACAAGGCATATCTAGGCGATGGTCTTGGCCCTTTGGAGTTTTTGACCAAGTTTCGG
      T  Y  K  A  Y  L  G  D  G  L  G  P  L  E  F  L  T  K  F  R  491
1682 ATACCGCATCCTGGAAGAGTTGGAGGAAGGTTTTTTGTTGACATGTTCATGCCTTCTATG
      I  P  H  P  G  R  V  G  G  R  F  F  V  D  M  F  M  P  S  M  511
1742 TTAAGTTGGGTCTTAGGTGGCAATAGCTCCAAACTTGAAGGCAGACCACTAAGTTGCAGA
      L  S  W  V  L  G  G  N  S  S  K  L  E  G  R  P  L  S  C  R  531
1802 CTCTCTGACAAAGCAAATGACCAGTTGCGTAGATGGTTTGAAGATGATGATGCACTTGAG
      L  S  D  K  A  N  D  Q  L  R  R  W  F  E  D  D  D  A  L  E  551
1862 CGTGCTAATAGTGGAGAGTGGACTTTATTACCACAAGGAGAAGAAACAGGTCATTCAAAA
      R  A  N  S  G  E  W  T  L  L  P  Q  G  E  E  T  G  H  S  K  571
1922 CCTATAAGTTTAAGTCGAGATGAGATGAAACCGTGCATGATCGGGAGTGCACAGGAAAAT
      P  I  S  L  S  R  D  E  M  K  P  C  M  I  G  S  A  Q  E  N  591
1982 GATTTTATTGGCAATTCAATAACAATACCTTCACCCCAGGTTTCTCCGAGACATGCTCGA
      D  F  I  G  N  S  I  T  I  P  S  P  Q  V  S  P  R  H  A  R  611
2042 ATTAGCTATAAGGATGGTGCCTTCTTCGTGACTGATTTGCGAAGTCAACATGGCACTTCG
      I  S  Y  K  D  G  A  F  F  V  T  D  L  R  S  Q  H  G  T  S  631
2102 ATCATTGACATTGAAGGAAGGAAATATCGGGTACCTCCAAATTATCCTGCTCAGGTCCAC
      I  I  D  I  E  G  R  K  Y  R  V  P  P  N  Y  P  A  Q  V  H  651
2162 CCAACCGATGTTATTGAGTTTGGCTCTGATAAGGCTTCATTTCGGGTTAAGGTTATCAGA
      P  T  D  V  I  E  F  G  S  D  K  A  S  F  R  V  K  V  I  R  671
2222 TCTGCACCAAGAGTATCTGAGAAGGAGGAAATGACAAAAGTTCCTGTGAAAGTATAAgtg
      S  A  P  R  V  S  E  K  E  E  M  T  K  V  P  V  K  V        690
2282 attatatttagttattaattacagtttgtaagtgttacagaaaagttgtacagcacaaat
2342 ttggaaaggtagagccactatcttccctttaaattgggtattatacaatagtgtcagtc
2402 ataaaggatatgaacatattacaaatttttaattgtgaatcatagaatgaaggtatatag
2462 ttgttgatataataggaaacaaagttaagtttttccg
```

**Supplementary Figure 5.** cDNA sequence of *LlZEP* and predicted amino acid LlZEP sequence. The positions of nucleotides and amino acids are marked on the left and right side, respectively. START and STOP codons are indicated by large, black, italics letters. UTR regions were highlighted before ATG and after TGA codons (small, blue letters). Predicted LlZEP sequence contains conserved domains for the zeaxanthin epoxidase protein family: the FAD/NAD(P)-binding domain (blue colored) containing FAD-binding motif (black squares) and FHA - Forkhead-associated domain indicated by yellow.

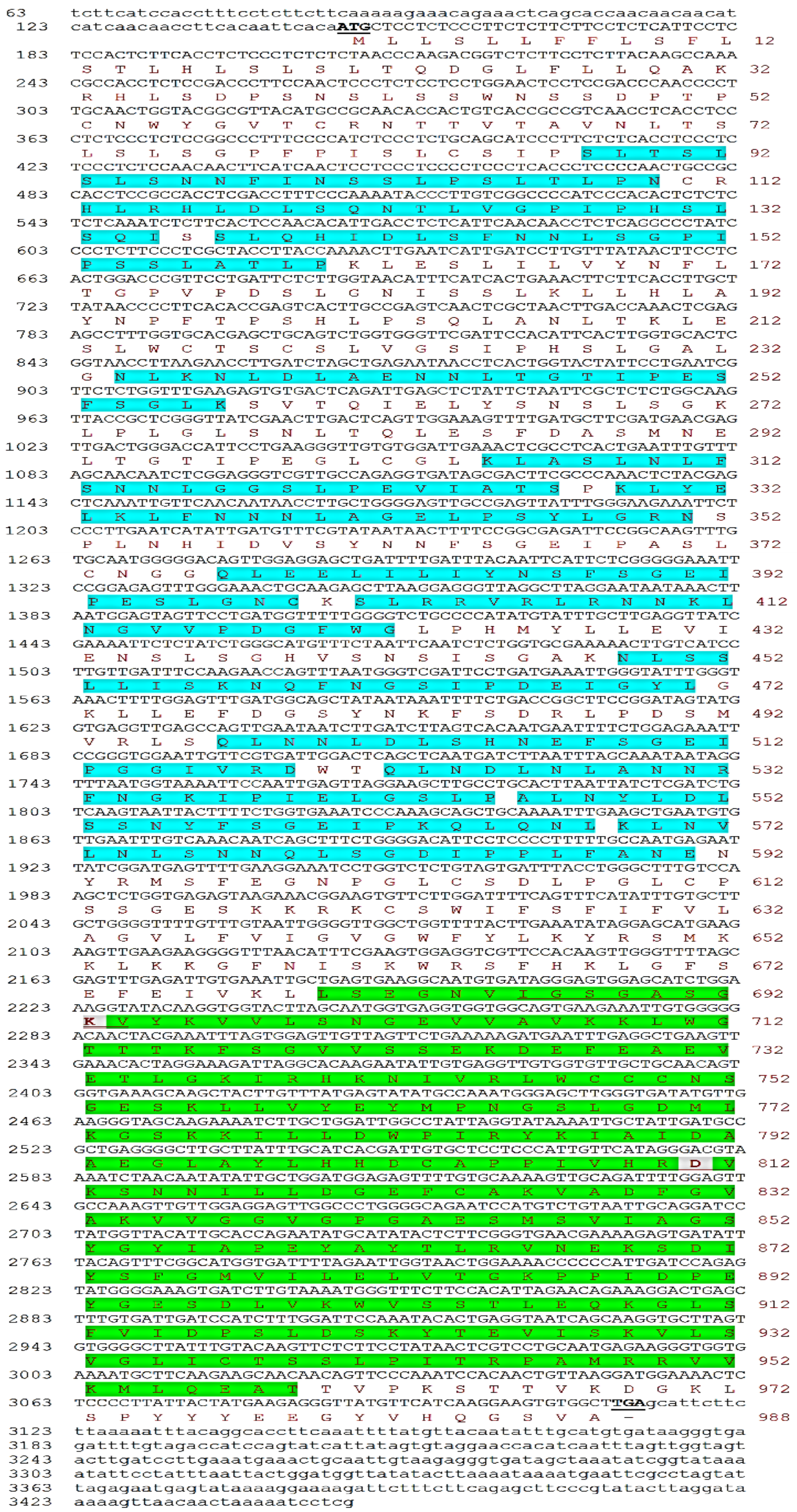

**Supplementary Figure 6.** cDNA sequence of *LlHSL* and predicted amino acid LlHSL sequence. The positions of nucleotides and amino acids are marked on the left and right side, respectively. START and STOP codons are indicated by large, black, italics letters. UTR regions were highlighted before ATG and after TGA codons (small, blue letters). Leucine-rich repeat (LRR) receptor motif characteristic for serine/threonine protein kinases responsible for protein-protein interactions is indicated by blue. The catalytic domain is marked by green. N-terminal domain is glycine-rich (underlined G) and contains lysine residue (K, grey indicated) determining ATP binding. The second region important for catalytic activity in the central part of the domain is underlined. It contains conserved aspartic acid (bolded and grey) (Knighton et al. 1991).

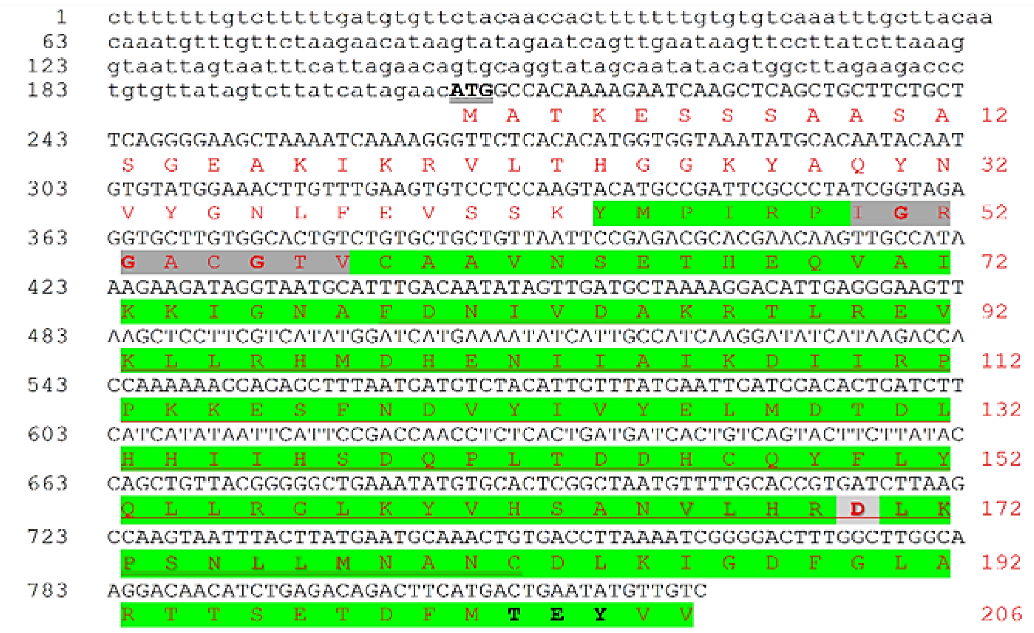


**Supplementary Figure 7.** The fragment of cDNA sequence of *LlMPK6* and predicted amino acid LlMPK6 sequence. The positions of nucleotides and amino acids are marked on the left and right side, respectively. START and STOP codons are indicated by large, black, italics letters. UTR regions were highlighted before ATG and after TGA codons (small, blue letters). Catalytic domain characteristic for serine/threonine and tyrosine protein kinases is indicated by green. N-terminal, glycine-rich (bolded G) fragment (indicated by grey) is involved in ATP binding. The second active region of the kinase is bolded. It contains conserved aspartic acid (grey D) determining enzymatic activity. The activation loop contains a characteristic TEY motif (bolded black letters) (Knighton et al. 1991).

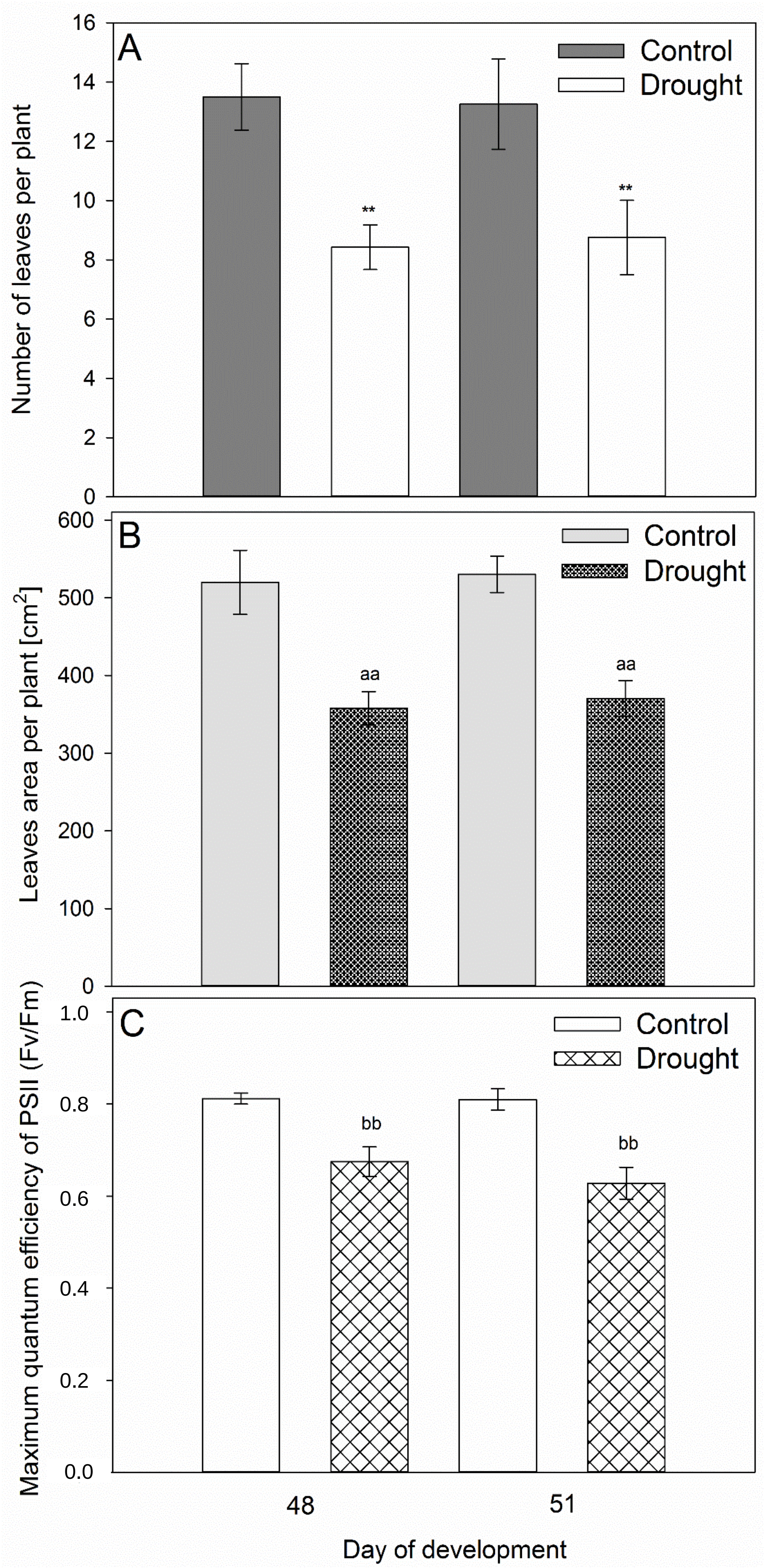


**Supplementary Figure 8.** The influence of soil drought stress on the number of leaves per plant (**A**), leaves area per plant [%] (**B**) and maximum quantum efficiency of PS II (**C**) in *Lupinus luteus* L. Control plants were growing in the soil of optimal moisture (70% WHC). In parallel, part of plants was subjected to drought conditions for 2 weeks (25% WHC). Measurements were performed on the 48th and 51st days of cultivation. The data were presented as averages of 15 technical replicates ± SE. Significant differences to stress plants in comparison to control are indicated as **P<0.01 (A), as $^{aa}$P<0.01 (B), as $^{bb}$P<0.01 (**C**), (t-Student test).

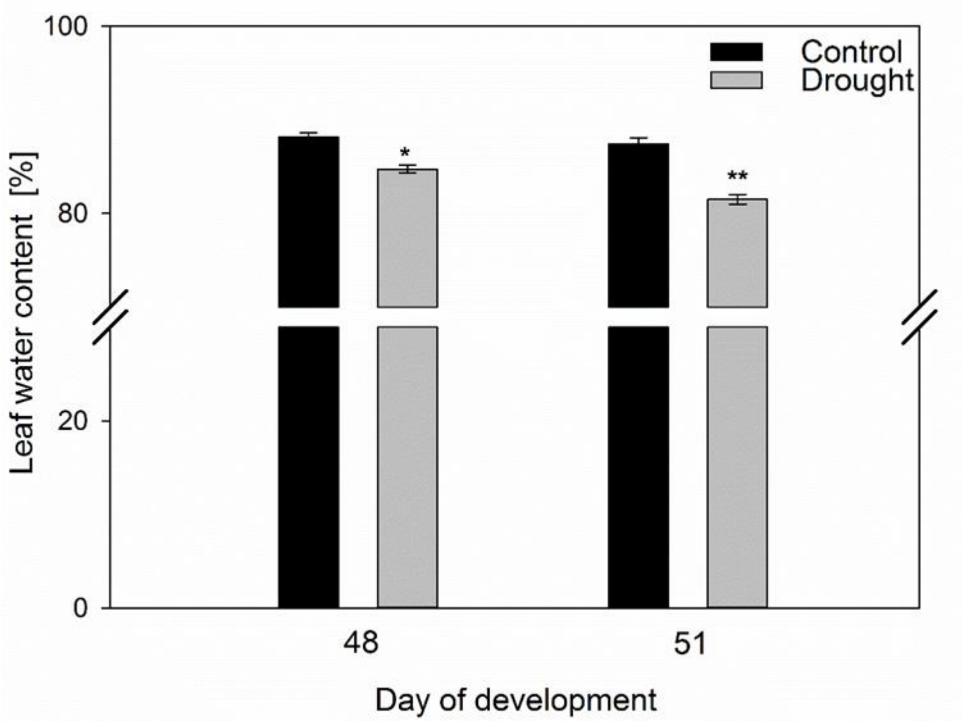


**Supplementary Figure 9.** The influence of soil drought stress on the water content in leaves [%] of *Lupinus luteus* L. Control plants were growing in the soil of optimal moisture (70% WHC). In parallel, part of plants was subjected to drought conditions for 2 weeks (25% WHC). Plant material for water content measurements was collected on the 48th and 51st days of cultivation. The data were presented as averages of 3 technical replicates ± SE. Significant differences to stress plants in comparison to control are indicated as *P<0.05, **P<0.01 (t-Student test).

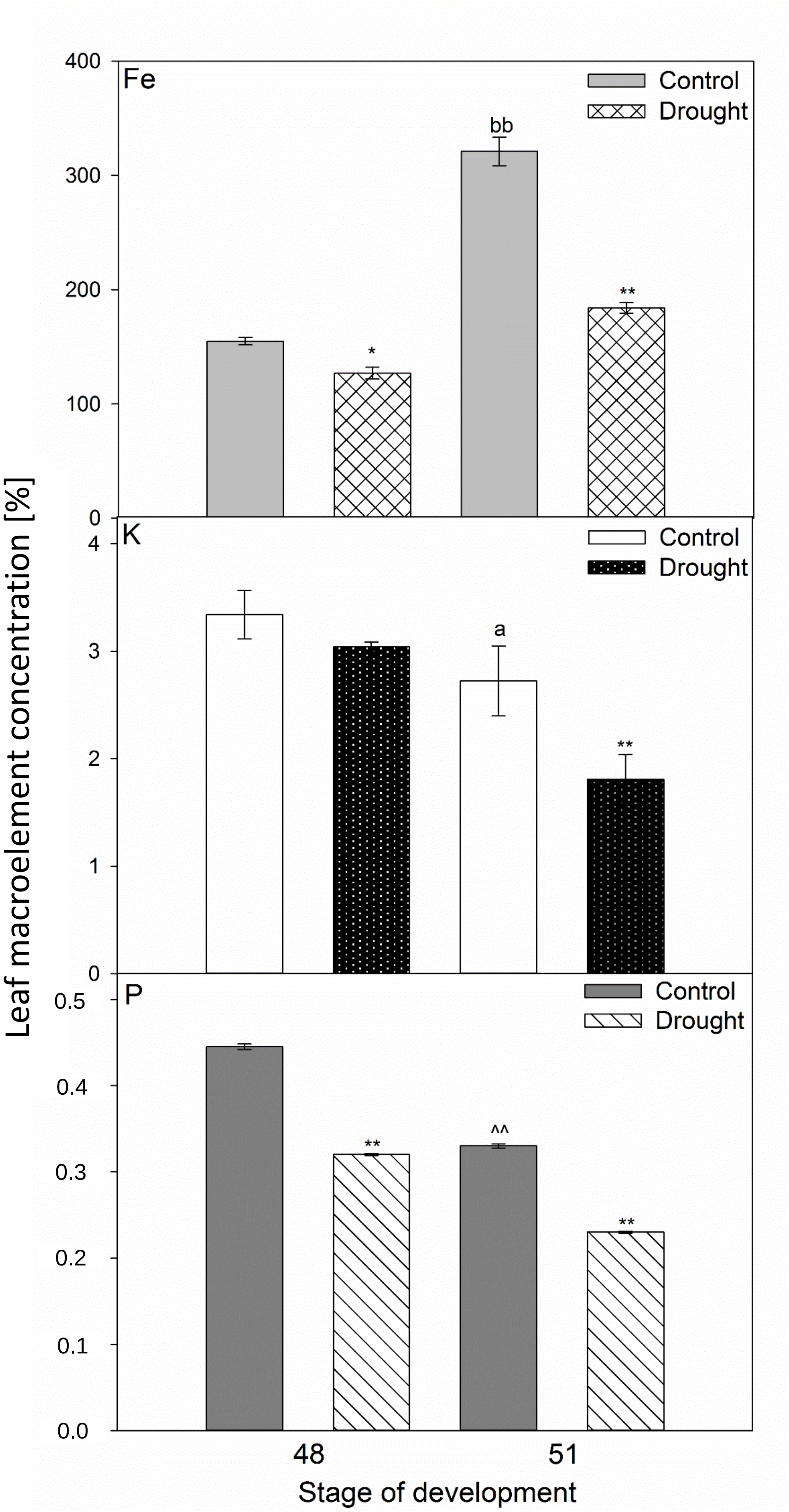


**Supplementary Figure 10.** The impact of soil drought on the elements content in leaves [%] of *Lupinus luteus* L. Control plants were growing in the soil of optimal moisture (70% WHC). In parallel, part of plants was subjected to drought conditions for 2 weeks (25% WHC). Material for measurements was collected on the 48th and 51st days of cultivation. The data were presented as averages of 3 technical replicates ± SE. Significant differences to stress plants in comparison to control are indicated as *P<0.05, **P<0.01 (for Fe); significant differences to stress plants in comparison to control are indicated as **P<0.01, significant differences to 51-days control plants in comparison to 48-day-old plants are indicated as $^{a}$P<0.01 (for K); significant differences to stress plants in comparison to control are indicated as **P<0.01, significant differences to 51-days control plants in comparison to 48-day-old plants are indicated as ^^P<0.01 (for P), (t-Student test).

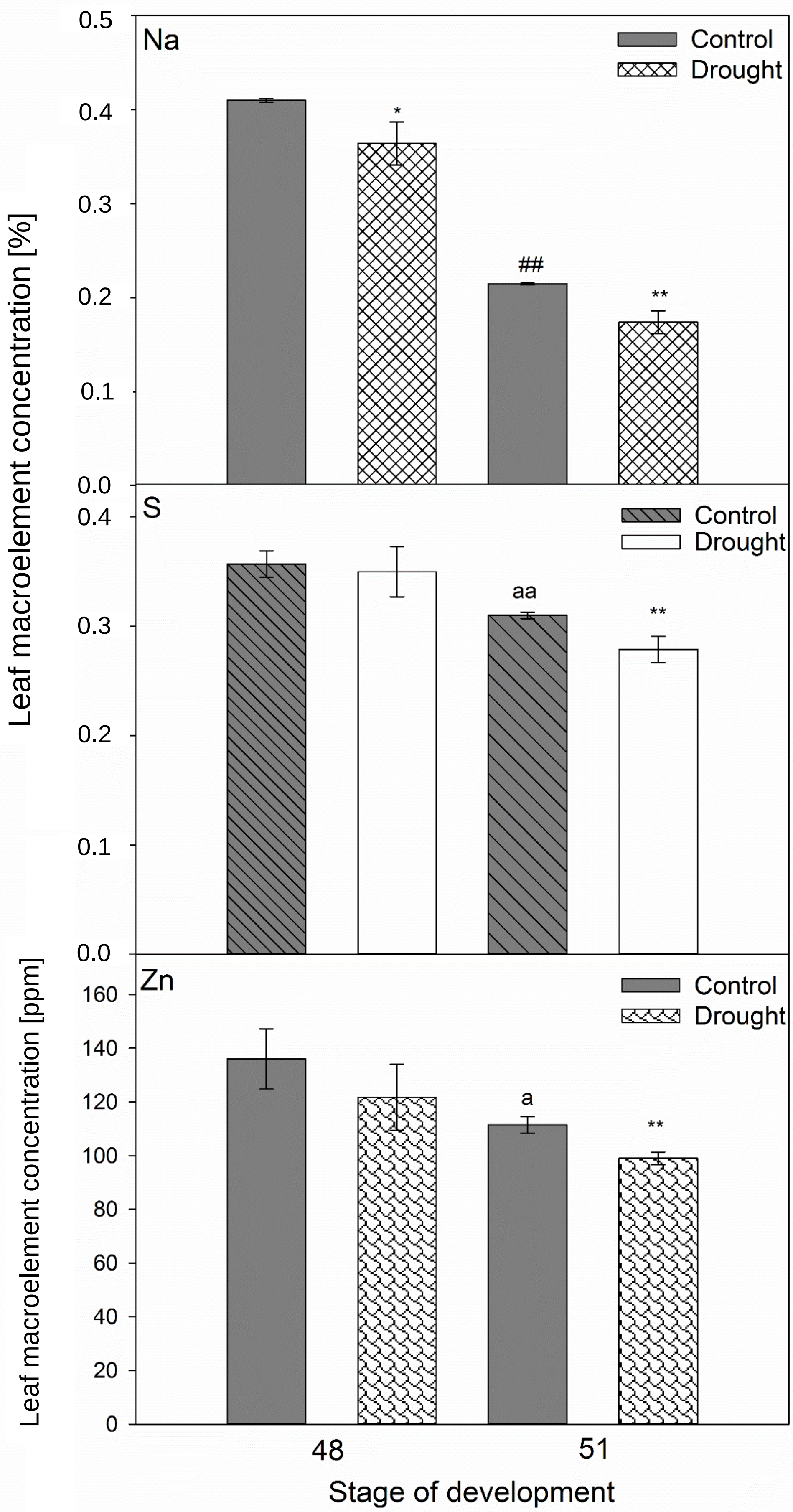


**Supplementary Figure 11.** Elements content in leaves [%] of yellow lupines cultivated under drought stress conditions. Control plants were growing in the soil of optimal moisture (70% WHC). Part of plants was subjected to drought conditions for 2 weeks (25% WHC). Material for water content measurements was collected on the 48th and 51st days of cultivation. The data were presented as averages of 3 technical replicates ± SE. Significant differences to stress plants in comparison to control are indicated as *P<0.05, **P<0.01, significant differences to stress plants in comparison to control are indicated as $^{\#\#}$P<0.01 (for Na); significant differences to stress plants in comparison to control are indicated as **P<0.01, significant differences to 51-days control plants in comparison to 48-day-old plants are indicated as $^{aa}$P<0.01 (for S); significant differences to stress plants in comparison to control are indicated as **P<0.01, significant differences to 51-days control plants in comparison to 48-day-old plants are indicated as $^{a}$P<0.05 (for Zn) (t-Student test).
